# Anatomic single vs. double-bundle ACL reconstruction: a randomized clinical trial–Part 1: clinical outcomes

**DOI:** 10.1007/s00167-021-06585-w

**Published:** 2021-05-10

**Authors:** James J. Irrgang, Scott Tashman, Charity G. Patterson, Volker Musahl, Robin West, Alicia Oostdyk, Bryan Galvin, Kathleen Poploski, Freddie H . Fu

**Affiliations:** 1 Department of Physical Therapy, School of Health and Rehabilitation Sciences, University of Pittsburgh, Suite 210, Bridgeside Point 1, 100 Technology Drive, Pittsburgh, PA 15203 USA; 2 Department of Orthopaedic Surgery, School of Medicine, University of Pittsburgh, Pittsburgh, PA USA; 3 Steadman Philippon Research Institute, Vail, CO USA; 4 UPMC Freddie Fu Sports Medicine, Center, Pittsburgh, PA USA; 5 Inova Sports Medicine, Fairfax, VA USA

**Keywords:** Anterior cruciate ligament reconstruction, Anatomic single-bundle, Anatomic double-bundle, Randomized clinical trial, Clinical outcomes

## Abstract

**Purpose:**

Compare clinical outcomes of anatomic single-bundle (SB) to anatomic double-bundle (DB) anterior cruciate ligament reconstruction (ACLR). It was hypothesized that anatomic DB ACLR would result in better International Knee Documentation Committee Subjective Knee Form (IKDC-SKF) scores and reduced anterior and rotatory laxity compared to SB ACLR.

**Methods:**

Active individuals between 14 and 50 years of age that presented within 12 months of injury were eligible to participate. Individuals with prior injury or surgery of either knee, greater than a grade 1 concomitant knee ligament injury, or ACL insertion sites less than 14 mm or greater than 18 mm were excluded. Subjects were randomized to undergo SB or DB ACLR with a 10 mm-wide quadriceps tendon autograft harvested with a patellar bone block and were followed for 24 months. The primary outcome measures included the IKDC-SKF and KT-1000 (side to side difference) and pivot shift tests. Other secondary outcomes included measures of sports activity and participation, range of motion (ROM) and re-injury.

**Results:**

Enrollment in the study was suspended due to patellar fractures related to harvest of the patellar bone plug. At that time, 57 subjects had been randomized (29 DB) and two-year follow-up was attained from 51 (89.5%). At 24-month follow-up there were no between-group differences detected for the primary outcomes. Twenty-one (77.8%) DB’s and 20 (83.3%) SB’s reported returning to pre-injury sports 2 years after surgery (n.s) Three subjects (2 DB’s, 5.3% of total) sustained a graft rupture and 5 individuals (4 SB’s, 8.8% of total) had a subsequent meniscus injury.

**Conclusions:**

Due to the early termination of the study, there were no detectable differences in clinical outcome between anatomic SB and DB ACLR when performed with a quadriceps tendon autograft with a bone block in individuals with ACL insertion sites that range from 14 to 18 mm.

**Level of Evidence:**

Level 2

**Supplementary Information:**

The online version contains supplementary material available at 10.1007/s00167-021-06585-w.

## Introduction

Anterior cruciate ligament reconstruction is generally perceived to successfully restore knee stability and enable individuals to return to their prior activity level. However, several meta-analyses [4, 5] concluded that ACLR fails to restore normal structure and function of the knee. Furthermore, only 65% of individuals return to their pre-injury level of sports participation and 55% of competitive athletes return to sports [2]. Perhaps more concerning is that ACLR does not appear to reduce the risk of PTOA after ACL injury. After ACLR, the prevalence of radiographic OA ranges from 39 to 90% 7–12 years after surgery [7, 16, 19].

Methods to anatomically reconstruct the ACL, in which the tunnels are placed within the anatomic footprints of the tibial and femoral insertions of the native ligament, have been proposed to improve the outcomes of ACL surgery. Anatomic methods to reconstruct the ACL include single-bundle (SB) ACLR, in which a single graft is used to replace both bundles of the ACL or double-bundle (DB) ACLR, in which separate grafts are used to replace each bundle of the ACL. It is believed that in comparison to SB ACLR, DB ACLR more closely restores normal structure of the knee, leading to more normal knee kinematics and improved clinical outcomes. Meta-analyses of level 1 and 2 studies comparing SB and DB ACLR [14, 15, 17, 18, 21, 28] have resulted in inconsistent conclusions, with some concluding that DB ACLR results in reduced rotational [14, 15, 18, 20, 28] and anterior laxity, [6, 15, 17, 18, 20, 28] however few have demonstrated a benefit in terms of patient-reported outcomes [17]. None of the meta-analyses included studies in which the same graft type and size was used for all individuals and in which the size and location of the insertion sites was controlled.

To address this gap in evidence, a double-blind randomized clinical trial was conducted to compare clinical outcomes of anatomic SB to anatomic DB ACLR. In this study, a 10-mm-wide quadriceps tendon autograft with bone block was utilized for all cases and only individuals that had insertion sites that ranged from 14 to 18 mm were included. It was hypothesized that anatomic DB ACLR would result in better International Knee Documentation Committee Subjective Knee Form (IKDC – SKF) scores and reduced side to side differences in laxity as measured with the KT-1000 and pivot shift tests compared to SB ACLR. Other secondary outcomes that were assessed included measures of sports activity, return to sports participation, range of motion and re-injury.

## Material and methods

This clinical trial was reviewed and approved by the University of Institutional Review Board for Biomedical Research (PRO) and registered on ClinicalTrials.gov (NCT). The Clinical Protocol for the clinical trial is included as Supplement 1.

### Subjects

Subjects were recruited from the clinical practices of between March 2011 and December 2012. Individuals with a complete tear involving both bundles of the ACL were eligible if they presented for surgery within 12 months of injury, were between 14 and 50 years of age, participated in at least 100 h of level 1 (e.g. football, basketball or soccer) or 2 (e.g. racquet sports, skiing, manual labour occupations) activities in the year prior to injury and had tibial and femoral ACL insertion sites widths between 14 and 18 mm. Individuals with injury to the medial and/or lateral meniscus were eligible for inclusion in the study. Individuals were excluded if they had prior injury or surgery of the ipsilateral or contralateral knee, greater than a grade 1 concomitant knee ligament injury, a full-thickness cartilage injury, open femoral or tibial growth plates, inflammatory or other forms of arthritis, any other injury or condition involving the lower extremity that affected the individual’s ability to participate in Level 1 or 2 activities or plans to move from the region within the study follow-up period. Females that were pregnant or with plans to become pregnant within two years were also excluded. Individuals were also excluded if the thickness of the quadriceps tendon on a sagittal MRI cut was less than 7 mm.

### Overview of research procedures

After obtaining informed consent, subjects participated in a pre-operative visit during which baseline demographics, pre-injury activity and participation and clinical outcome measures were collected. After an examination under anaesthesia and diagnostic arthroscopy to confirm final eligibility, subjects were randomized to undergo anatomic DB or SB ACLR. Follow-up occurred at 3, 6, 12- and 24-months following randomization. Primary outcome measures included the IKDC-SKF and KT-1000 (side to side difference) and pivot shift tests. Other secondary outcomes that were assessed included measures of sports activity and participation, return to sports participation, range of motion (ROM) and re-injury.

### Examination under anesthesia and diagnostic arthroscopy

Subjects that met the preliminary eligibility criteria were scheduled for standard of care diagnostic arthroscopy and ACLR. Following induction of anaesthesia, an examination under anaesthesia (EUA) and diagnostic arthroscopy were performed to ensure that subjects met all final eligibility criteria including disruption of both bundles of the ACL and tibial and femoral insertion sites were between 14 and 18 mm as measured by an arthroscopic ruler.

### Randomization procedures

The randomization list was generated by the study biostatistician (CP) using permuted blocks with random blocks sizes in SAS, stratified based on surgeon, subject age (14–23, 24–50 years of age), sex and meniscus status. Group assignments were concealed until final eligibility of each participant was confirmed during the EUA and diagnostic arthroscopy. To minimize bias throughout the study, both the subject and research assistant responsible for collecting the outcome measures remained blinded to group assignment.

### Surgical reconstruction of the ACL

**Fig. 1:**
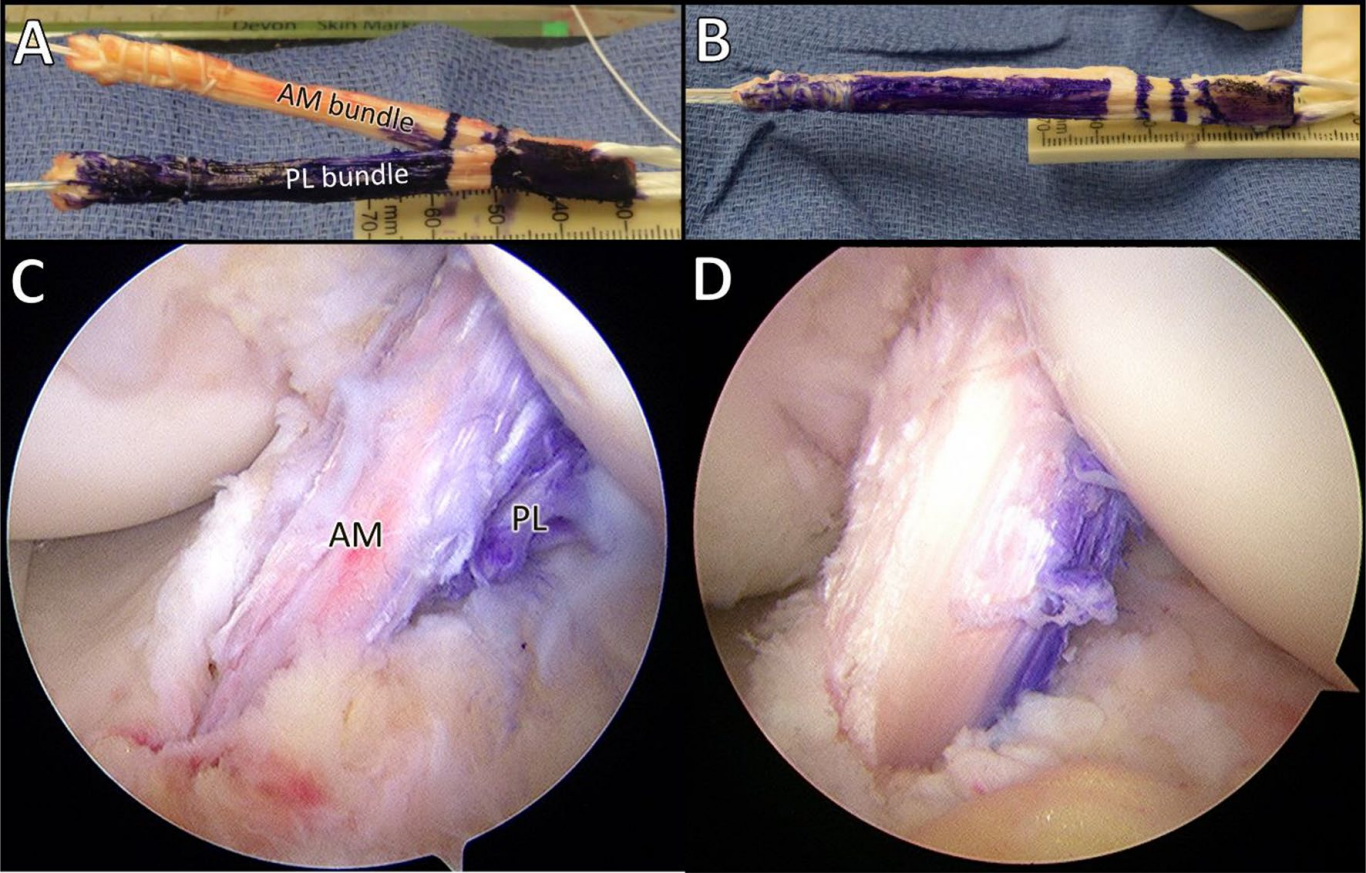
**a** Split quadriceps tendon graft with bone block for anatomic double bundle ACL reconstruction; **b** Quadriceps tendon graft with bone block for single-bundle ACL reconstruction; **c** Arthroscopic appearance of anatomic double-bundle and **d** Arthroscopic appearance of anatomic single-bundle ACL reconstruction (*AM *anteromedial bundle of reconstructed ACL, *PL *posterolateral bundle of reconstructed ACL)

All surgeries were performed using standardized procedures for anatomic ACLR. The procedures for anatomic DB ACLR and SB ACLR have been published [25]. To avoid graft type as a confounding factor, a 10 mm-wide autograft quadriceps tendon with a patellar bone block was used in all cases [8]. For DB ACL reconstruction, the 10 mm-wide quadriceps tendon graft was split, leaving the bone block as one, into grafts to reconstruct the anteromedial (AM) and posterolateral (PL) bundles (see Fig. 1a and b). To minimize bias with graft harvest, the harvest was completed before the subject was randomized into the SB or DB group. For DB ACLR, one femoral tunnel in the center of the femoral insertion site and two tibial tunnels corresponding to the insertions of the AM and PL bundles were created to reproduce the normal insertion site anatomy. For SB ACLR, one femoral and one tibial tunnel were created in the center of the femoral and tibial insertion sites respectively (see Fig. 1c and d). If necessary, meniscus repair or meniscectomy or chondroplasty was performed.

### Post-operative rehabilitation

All subjects underwent the same standardized post-operative rehabilitation program, supervised by a physical therapist, as previously described by Irrgang and Enseki [13].

### Assessment of clinical outcomes

Clinical outcomes, including patient-reported outcomes, return to sports, laxity and ROM were measured before surgery and 3, 6, 12 and 24 months after surgery. A single trained clinical research assistant (BG), who was blinded to the subject’s group assignment administered questionnaires and performed all laxity and ROM tests.

### Patient-reported outcomes

The primary patient-reported outcome was the IKDC – SKF, which is a measure of symptoms, function and sports activities for individuals with a variety of knee conditions, including ACL injuries. The IKDC – SKF has undergone extensive psychometric testing [12] and the threshold for the patient acceptable symptom state after ACLR [24] has been determined. Secondary patient-reported outcomes included the Activities of Daily Living Scale of the Knee Outcome Survey (KOS – ADLS), Knee injury and Osteoarthritis Outcome Scale (KOOS), Veteran’s RAND 12 Item Health Survey (VR-12), Activity Measure for Post-Acute Care (AM-PAC), and the ACL Return to Sports after Injury Scale (ACL-RSI).

### Knee joint laxity and range of motion

The primary outcomes for knee laxity were tibial rotation and anterior translation as measured with the pivot shift test and the KT-1000 Knee Arthrometer (MedMetric Inc., San Diego, CA), respectively. Anterior translation was measured at 25° of knee flexion with a 134 N anterior load. Because intra-rater reliability for measurement of anterior translation with the KT-1000 is high (ICC = 0.90–0.99), but inter-tester reliability is lower (ICC = 0.61–0.82), all testing was performed by a single trained clinical research assistant. The Lachman test served as a secondary measure of laxity.

The side-to-side difference in passive knee extension and flexion, measured in the supine position with a standard goniometer served as a secondary outcome measure. Intra- and inter-tester reliability coefficients are 0.98 and 0.86 for passive knee extension and 0.99 and 0.90 for knee flexion.

All measures of laxity and range of motion were compared side-to-side and graded according to the IKDC Knee Ligament Rating guidelines (normal, nearly normal, abnormal, severely abnormal). For analysis, all variables were then dichotomized as normal (side-to side differences of  – 1 to 2.5 mm for anterior tibial translation, equal for pivot shift test, < 3 mm for Lachman’s test, side-to-side differences < 3° for extension, or < 5º for flexion) vs. nearly normal or worse classifications.

### Return to sports

Sports activity was measured with the Marx Activity Scale (MAS). The time reference for the subject to report his or her activity level when completing the MAS after surgery was modified to the prior month at three and six months after surgery, the prior three months at twelve months after surgery and the prior year (unmodified) for the year prior to injury and the 24-month follow-up. The type and frequency of sports participation were assessed before injury using scales modified from the Cincinnati Knee Rating Scale and the IKDC – SKF. Finally, a global question on return to sports “Since your surgery, have you returned to the same sports that you participated in before injury?” with the response of yes or no was also answered by patients.

### Statistical analysis

All analyses were conducted using intention to treat. First, the distribution of baseline characteristics and important prognostic factors (age, sex, pre-injury activity level, meniscus injury/surgery, BMI, etc.) were compared between the DB and SB groups to assess the effectiveness of randomization.

The primary patient-reported outcome was the IKDC – SKF, which was treated as a continuous variable that was measured at four time points (baseline, 6, 12 and 24 months). As such, mixed effects models for repeated measures were utilized treating group (2 levels), time (3 levels), and the group by time interaction as fixed effects and controlling for intra-patient correlation using a subject specific random effect.

The primary outcomes for assessment of laxity were the pivot shift and the side to side difference for the KT-1000. Both measures were collected at 3, 6, 12 and 24 months after surgery, but the primary endpoint was the 24-month value. For the pivot shift test, the four-level score was collapsed to create a binary variable (normal vs. not normal [nearly normal, abnormal, severely abnormal]). Fisher’s exact tests were used to compare the proportion of patients with a “normal” pivot shift test between the DB and SB ACLR groups. A two-sample t-test was used to test the mean side to side difference between groups for the KT-1000 test at 24 months.

Similar analyses to those described for the primary outcomes above, were performed for the secondary outcomes to support our primary findings. The secondary patient-reported outcomes included the KOS – ADLS, 5 scales of the KOOS, VR-12, AM-PAC and ACL-RSI. Other secondary measures included the MAS and return to sports as well as measures of knee joint function including the Lachman test and side to side differences in ROM. For these analyses, Hochberg’s step-up procedure was used to adjust for multiple secondary endpoints (α = 0.10) within each specific hypothesis that was tested.

### Sample size determination

In planning the study, it was specified that DB ACLR would be considered superior to SB ACLR if the IKDC – SKF score was 12 points greater (which is approximately equal to the minimal detectable change and minimum clinically important difference [12], the proportion of normal pivot shift tests was 20% greater and/or the side to side difference for the KT-1000 was 1.5 mm less in comparison to SB ACLR. It was determined that a total sample size of 136 subjects (68 per group), would provide greater than 80% power to detect a 7-point difference in the IKDC – SKF, assuming a common standard deviation among groups of 14 with a two tailed *t*-test with alpha of 0.05. This sample size also enabled detection of a 20% difference in the proportion of normal pivot shift tests assuming the proportion of normal pivot shift tests in the SB ACLR group was 70% (based on a meta-analysis by Meredick et al. [21]) and 1.2 mm side to side difference in the KT-1000 assuming a common standard deviation of 2.2 mm (based on the pooled standard deviation from a meta-analysis by Meredick et al. [21]) with two-tailed tests with alpha of 0.025 to account for the multiple comparisons for this hypothesis. To account for a 15% loss of follow-up at two years, our target enrollment of the study was therefore 160 subjects.

### Data and safety monitoring

Adverse events were monitored continuously throughout the study and were reviewed by the investigators and an independent Data and Safety Monitoring Board (DSMB) to determine severity and relationship with the research intervention and procedures. No formal interim analyses were conducted during the trial.

### Participant recruitment

**Fig. 2:**
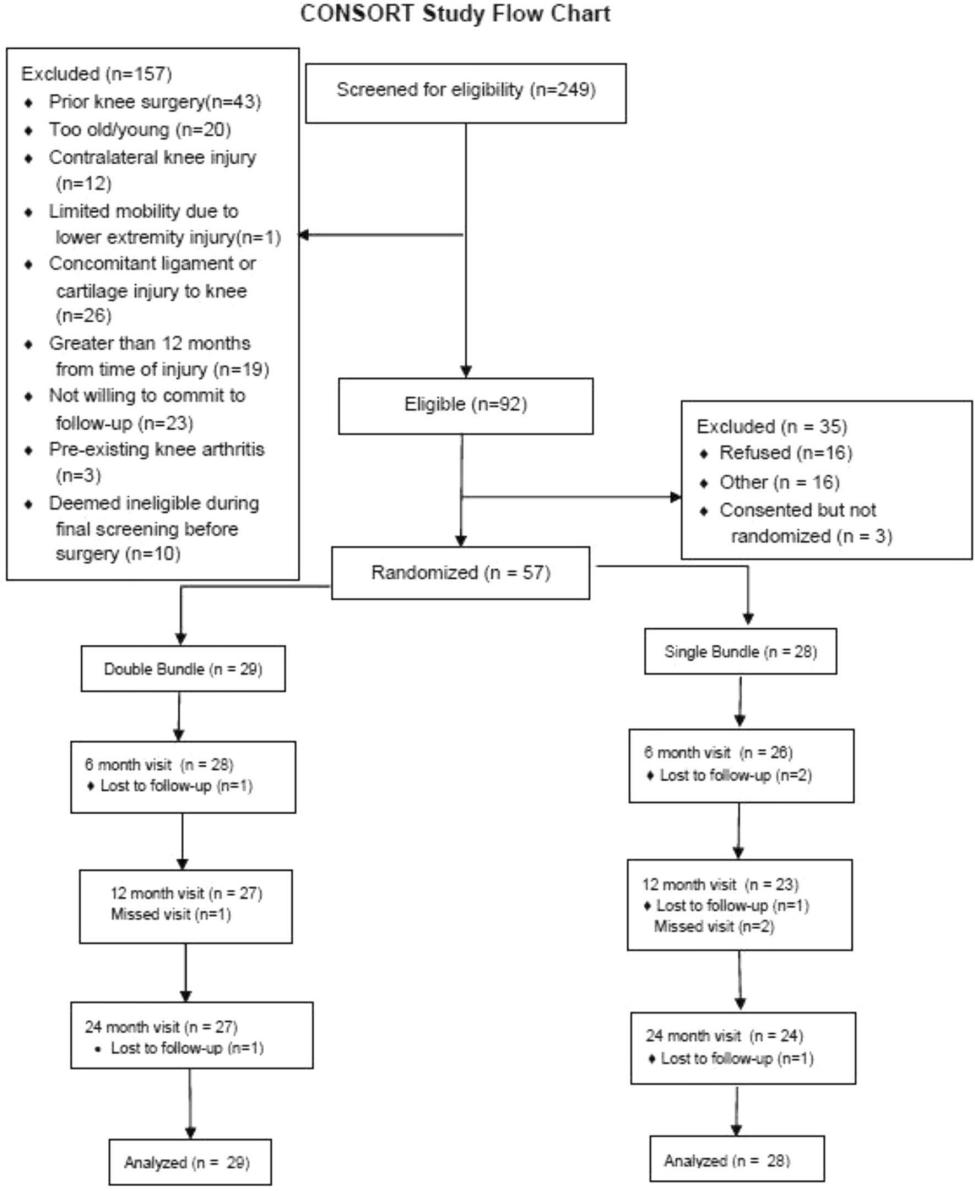
CONSORT study flow chart

Between March 2011 and December 2012, 249 individuals with a suspected ACL injury were screened to determine eligibility for participation in the study. The details of recruitment and follow-up are provided in the CONSORT Study Flow Chart in Fig. 2. A total of 57 subjects were randomized to SB (*n* = 28) or DB (*n* = 29) ACLR.

In December 2012, enrollment in the study was permanently suspended by the DSMB due to the occurrence of five patellar fractures related to harvest of the patellar bone plug (see Adverse Events section for further details). After enrollment was suspended, the DSMB approved continued follow-up of the enrolled subjects through the two-year endpoint. Six patients (2 DB, 4 SB ACLR) were lost to follow-up resulting in an overall follow-up rate of 89.5%.

## Results

### Subject characteristics

Baseline demographic, pre-injury activity and clinical findings of the subjects are summarized in Table 1. Groups were comparable on age, sex, weight, height, body mass index, race or ethnicity. Type and frequency of sports participation and the MAS scores prior to injury were similar in both groups as were concomitant meniscal procedures during surgery.

**Table 1:** Baseline demographic, pre-injury activity and clinical findings of study participants

	Double bundle (*n* = 29)	Single Bundle (*n* =28)
Age (years, mean ± SD)	23.1 ± 9.2	20.3 ± 4.3
Male (*n*, %)	18, 62.1%	20, 71.4%
Weight (lbs, mean ± SD)	170.8 ± 28.2	167.5, 28.1
Height (inches, mean ± SD)	69.1 ± 3.4	68.9 ± 3.6
Body mass index (kg/m^2^, mean ± SD)	25.1 ± 3.4	24.7 ± 2.7
*Race*		
Caucasian (*n*, %)	26, 89.7%	26, 92.9%
African-American (*n*, %)	3, 10.3%	2, 7.1%
Hispanic ethnicity (*n*, %)	0, 0.0%	0, 0.0%
*Frequency of sports activity*		
Competitive (4–7 times/week) (*n*, %)	24, 82.8%	21, 75.0%
Recreational (1–3 times/week) (*n*, %)	4, 13.8%	7, 25.0%
Non-Athlete (*n*, %)	1, 3.5%	0, 0.0%
*Type of sports*		
Very Strenuous (*n*, %)	23, 79.3%	25, 89.3%
Strenuous (*n*, %)	4, 13.8%	2, 7.1%
Moderate (*n*, %)	2, 6.9%	1, 3.6%
Marx activity scale (mean ± SD)	13.1 ± 4.5	14.4 ± 2.6
*Lachman test*		
–1 to 2 mm	2 (6.9%)	1 (3.6%)
3–5 mm (1 +)	23 (79.3%)	20 (71.4%)
6–10 mm (2 +)	4 (13.8%)	7 (25.0%)
*Pivot shift test*		
Equal	5 (17.2%)	5 (17.9%)
Glide (1 +)	18 (62.1%)	19 (67.9%)
Clunk (2 +)	6 (20.7%)	4 (14.3%)
KT – 1000 (134 N)	3.4 ± 2.1	3.6 ± 1.5
KT – 1000 (maximum manual)	3.8 ± 2.1	4.5 ± 1.6
*Concomitant surgical procedures*		
Meniscectomy (*n*, %)	5, 17.2%	2, 7.1%
Meniscus Repair (*n*, %)	4, 13.8%	6, 21.4%
Abrade & Trephination (*n*, %)	4, 13.8%	0, 0.0%

### Patient-reported outcomes

**Fig. 3:**
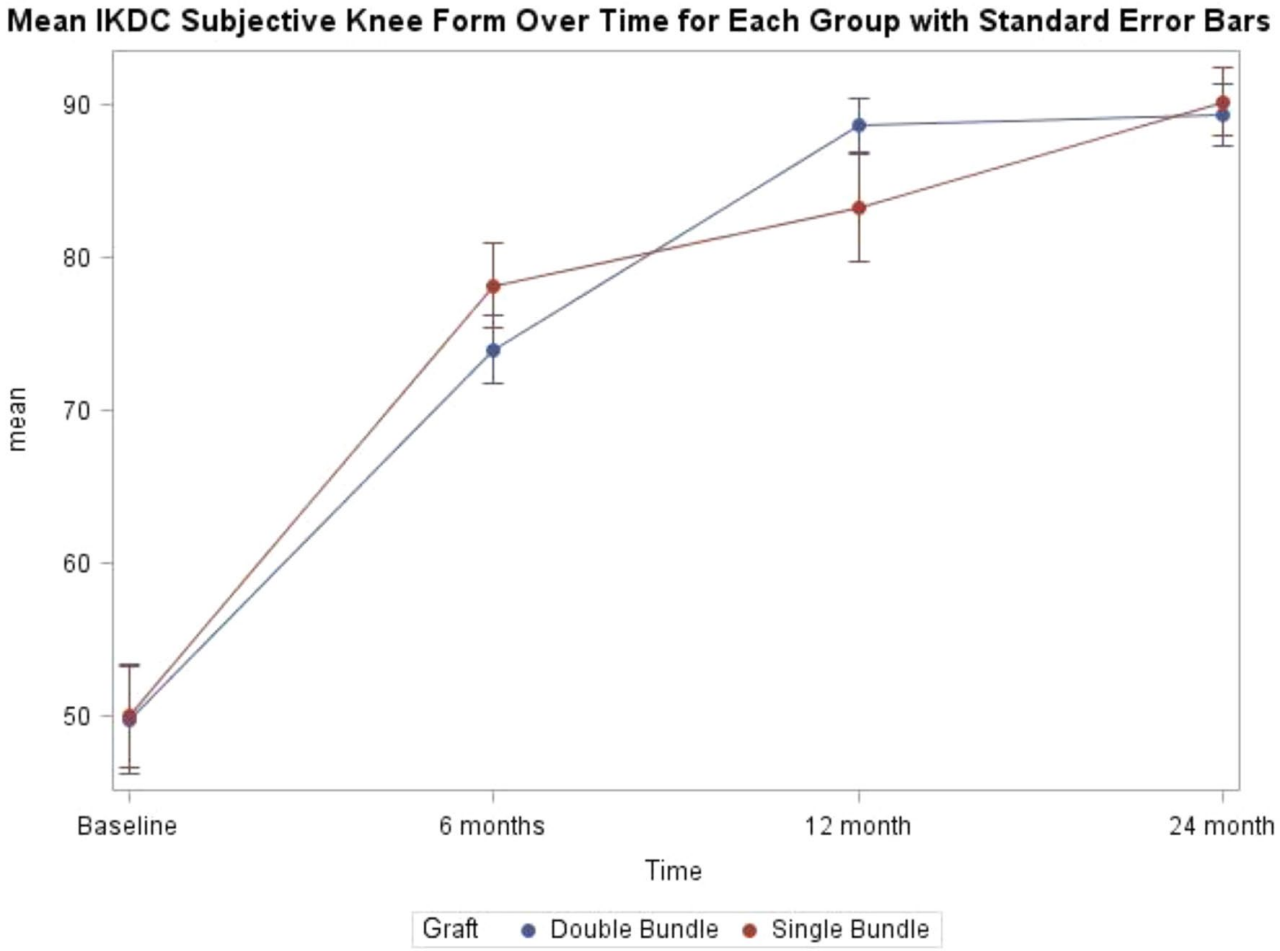
Mean IKDC subjective knee form scores over time with standard error bars for those that underwent single-bundle versus double-bundle ACL reconstruction. ^*a*^*P *= 0.43 for test of group by time interaction (fixed effects) controlling for intra-patient correlation (random effect)

No between group differences in the IKDC–SKF over time were detected by us (group by time interaction n.s., Fig. 3). At the 24 month follow-up, across both groups 94.4% of subjects achieved an IKDC – SKF that was within one standard deviation of the age- and sex-matched population average normative values [1] and 92.4% exceeded the threshold for the patient acceptable symptom state. [24] Generally there were no significant or clinically meaningful between group differences in the secondary patient-reported outcome measures at each follow-up time point (see supplemental Table 1). The exception to this was the difference in the VR-12 Physical Components Summary Scale (*p* = 0.02 for the test of interaction in the model) where the DB group had a lower 3-month average but relatively the same average at 24 months.

### Laxity and range of motion

Laxity at final follow-up of 24 months is summarized in Table 2. There were no significant between group differences in the side-to-side difference for the KT-1000 test at 134 N or the pivot shift and Lachman tests. The average side to side difference for the 134 N KT-1000 test was 0.5 ± 1.3 mm and 0.6 ± 1.6 mm for those undergoing DB and SB ACLR, respectively. Overall, 48 (94.1%) had a normal (equal) pivot shift test. Generally, there were no between group differences in the side-to-side differences in range of motion or the proportion of individuals that achieved normal knee extension (< 3° side to side difference) or flexion (< 5° side to side difference) at any follow-up time point (see Supplementary Table 2). Non-clinically relevant exceptions to this were the side-to-side difference in knee extension at 3 months, involved knee extension at 12 months and the 12-month side-to-side difference in knee flexion.

**Table 2:** Laxity at 24 month follow-up

	Double bundle (*n*=27)	Single bundle (*n*=24)	*P* value
KT-1000 (134 N)	0.5 ± 1.3	0.6 ± 1.6	n.s.^*a*^
*Pivot shift test*			
Equal (normal)	25 (92.6%)	23 (95.8%)	n.s.^*b*^
Glide (Nearly normal)	2 (7.4%)	1 (4.2%)	
Clunk (Abnormal)	0 (0.0%)	0 (0.0%)	
*Lachman test*			
– 1 to 2 mm (Normal)	24 (88.9%)	21 (87.5%)	n.s.^*b*^
3–5 mm (Nearly normal)	3 (11.1%)	2 (8.3%)	
6–10 mm (Abnormal)	0 (0.0%)	1 (4.2%)	

^*a*^Two-sample *t*-test used to compare mean side to side difference between DB and SB ACLR groups

^*b*^Fisher exact test used to compare proportion of patients with “normal” categorization between DB and SB ACLR groups

### Return to sports

There were no differences in the Marx Activity Scores across time (Table 3). Based on the global question of return to sports participation, 12 months after surgery 66.7% of those undergoing DB ACLR and 65.2% of those undergoing SB ACLR (n.s.) reported they were participating in the same sports that they were participating in prior to injury. At 24 months, 77.8% and 83.3% of those undergoing DB and SB ACLR (n.s.), respectively reported return to their prior injury sports participation.

**Table 3:** Sports activity

	Double bundle (*n*=29)	*n*	Single bundle (*n*=28)	*n*	*P* value
*Marx activity scale*					
3 Months	6.4 ± 7.6	26	6.4 ± 6.5	24	n.s.^*a*^
6 Months	5.8 ± 4.9	27	5.9 ±5.02	25	
12 Months	11.9 ± 4.9	27	12.8 ± 3.6	23	
24 Months	12.6 ± 4.2	27	11.5 ± 3.8	24	
*Returned to sports participation (Based on global question)*					
12 Months	18 (66.7%)		15 (65.2%)		n.s
24 Months	21 (77.8%)		20 (83.3%)		n.s

^*a*^ *P* value for test of group by time interaction (fixed effects) controlling for intra-patient correlation (random effect)

### Graft failures, additional surgical procedures and adverse events

The graft failures, additional surgical procedures and adverse events for those undergoing DB and SB ACLR are summarized in Table 4. Three individuals (5.3% of total) sustained a tear of the ACL graft, two in the DB ACLR and one in the SB ACLR group. Two individuals, both in the DB ACLR group elected to undergo revision ACLR. Four (7.0% of total) individuals sustained a contralateral ACL injury during the two-year follow-up period.

**Table 4:** Surgical failures, additional surgical procedures and adverse events

	Double bundle (*n* = 29)	Single bundle (*n* = 28)
Recurrent episodes of instability	2 (6.9%)	0 (0.0%)
Abnormal anterior laxity ≥ 6 mm	0 (0.0%)	0 (0.0%)
Abnormal rotatory laxity (clunk or gross pivot shift)	2 (6.9%)	2 (7.1%)
MRI evidence of graft failure	2 (6.9%)	1 (3.6%)
ACL revision	2 (6.9%)	0 (0.0%)
Other knee surgery	1 (3.5%)	5 (17.9%)
*Adverse events*		
Re-Tear of ACL Graft	2 (6.9%)	1 (3.6%)
Meniscus tear in surgical knee	1 (3.5%)	4 (14.3%)
Patellar fracture	0 (0.0%)	3 (10.7%)
Asymptomatic patellar fracture on research CT Scan	2 (6.9%)	0 (0.0%)
Loss of motion/arthrofibrosis	2 (6.9%)	0 (0.0%)
Cyclops lesion	0 (0.0%)	1 (3.6%)
Knee sprain	1 (3.4%)	0 (0.0%)
Quadriceps weakness	0 (0.0%)	1 (3.6%)
Patellar pain	1 (3.4%)	0 (0.0%)
Unspecified knee pain	3 (10.3%)	3 (10.7%)
Fall	2 (6.9%)	1 (3.6%)
Suture abscess	2 (6.9%)	4 (14.3%)
Infection	0 (0.0%)	0 (0.0%)
Pulmonary embolism	0 (0.0%)	0 (0.0%)
Nerve injury/paralysis	0 (0.0%)	0 (0.0%)
Vascular injury	0 (0.0%)	0 (0.0%)
Contralateral ACL tear	2 (6.9%)	2 (7.1%)
Contralateral meniscus tear	1 (3.4%)	0 (0.0%)
Contralateral popliteal cyst	1 (3.4%)	0 (0.0%)
Contralateral non-specific knee pain	1 (3.4%)	0 (0.0%)
*Serious adverse events*		
Deep vein thrombosis	2 (6.9%)	1 (3.6%)
Open reduction internal fixation for patellar fracture	0 (0.0%)	1 (3.6%)

Five (8.8% of total) individuals sustained a subsequent meniscus tear, 1 in the DB group and 4 in the SB group. Five of the individuals with a meniscus tear underwent surgical repair and/or meniscectomy.

Five subjects sustained a patellar fracture, two during harvest of the patellar bone block, one during isometric quadriceps strength testing at six months, and two asymptomatic occult patellar fractures were found on research-related computerized tomography (CT) scans six months after surgery. All patellar fractures healed and four of the individuals experienced no adverse symptoms or functional limitations through the two-year follow-up. One individual reported discomfort with prolonged standing and had an IKDC-SFK score of 64.4 at 24-month follow-up. Details of these cases are reported elsewhere [8].

## Discussion

The most important finding of the study was that due to the early termination of the study, there were no detectable differences in clinical outcome between anatomic SB and DB ACLR when performed with a quadriceps tendon autograft with a bone block in individuals with ACL insertion sites that range from 14 to 18 mm. Both SB and DB anatomical ACLR led to a high rate of excellent outcomes and return to sports participation by 24 months. The observed differences between the groups for the primary outcomes at 24 months were all small and not clinically meaningful.

To minimize the effects of graft size, a 10 mm-wide quadriceps tendon graft with a patellar bone block was utilized for all subjects that was harvested before subjects were randomized to SB or DB ACLR. The only differences in ACLR between the groups was that for DB ACLR, the graft was split into two arms and two rather than one anatomically placed tibial tunnels were created. Additionally, during DB ACLR, the PL graft was fixed with the knee at 0° of flexion and the AM graft was fixed at 45° of flexion, in comparison to fixation of the SB graft with the knee at 20° of flexion. Given these differences in the surgical procedures for DB and SB ACLR, no statistical or clinically meaningful differences were detected between the two surgical approaches for ACLR in individuals with insertion site sizes that range from 14 to 18 mm with no concomitant ligament injuries.

A randomized clinical trial involving 320 individuals with a torn ACL to compare conventional SB ACLR with anatomic SB or anatomic DB ACLR indicated that both anatomic SB and anatomic DB ACLR were superior to conventional SB ACLR [11]. In addition, there were no differences in outcome when the decision to perform SB or DB ACLR was based on the length of the ACL insertion site [10]. Therefore, given our current findings combined with the above findings [10, 11] we recommend individualized ACLR in which individuals with insertion sites less than 14 mm undergo anatomic SB ACLR and those with insertion sites greater than 18 mm undergo anatomic DB ACLR. Either anatomic SB or DB can be performed for individuals with insertion sites between 14 and 18 mm.

In general, the observed clinical outcomes for both anatomic SB and anatomic DB ACLR with a 10 mm-wide quadriceps tendon bone block are comparable or superior to the results reported for conventional ACLR [7–9, 22, 23, 26, 27]. Across both groups, at 24-month follow-up the average IKDC – SKF scores were on the order of 90, 94.4% of the subjects achieved an IKDC – SKF score that was within one standard deviation of their age- and sex-matched population average [1] and 92.4% of the subjects had a score that exceeded the patient acceptable symptom state for individuals one to five years after ACLR [24]. Additionally, the average side to side difference for the KT-1000 was on the order of 0.5–0.6 mm across all subjects and 94.1% had normal (< 3 mm side to side difference) anterior translation. For rotatory knee laxity, 94.1% had a normal (equal) pivot shift test. At two-year follow-up, the graft re-rupture rate was 5.3% and the meniscus injury rate was 8.8% across all subjects, are both within the ranges published for ACLR and were similar for both groups.

At the 12-month follow-up 64.7% of subjects reported they had returned to participation in their pre-injury sports and by the 24-month follow-up, participation in pre-injury sports increased to 80.4%. For comparison, a meta-analysis by Ardern et al. [3] reported 65% (95% CI 59–72%) returned their pre-injury level of sport.

The most concerning adverse events were the patellar fractures that were associated with harvest of the patellar bone block for the quadriceps tendon graft. Analysis of the study participants with a patellar fracture indicated the fracture was associated with harvest of the bone plug from the lateral portion of the patella and when the depth of the harvest was greater than 50% of the depth of the patella. We have since modified and described the procedures to harvest the quadriceps tendon grafts with a bone block to minimize the risk of patellar fracture [8].

This study is not without limitations. The primary limitation of this study was the occurrence of five patellar fractures that caused the DSMB to recommend suspending further enrollment in the study. Because of this, we were only able to recruit and randomize a total of 57 subjects, which was 41.9% of the subjects required to achieve 80% power as detailed in our sample size analysis. This limitation in sample size and power was partially offset by our achievement of follow-up rates approaching 90% at each time point. Other strengths of the study included concealment of randomization until final eligibility was determined at the time of the examination under anesthesia and diagnostic arthroscopy and harvest of the graft, blinding of both the patient and the research assistant that was responsible for collection of all outcome measures and use of a comprehensive set of clinical outcome measures.

## Conclusion

Due to the early termination of the study, there were no detectable differences in clinical outcome between anatomic SB and DB ACLR when performed with a quadriceps tendon autograft with a bone block in individuals with ACL insertion sites that range from 14 to 18 mm. The observed differences between groups at 24-month follow-up were small for all primary outcomes and not clinically relevant. Additionally, both anatomic SB and DB ACLR led to clinical outcomes that were comparable or superior to those reported for non-anatomic ACL reconstruction with minimal recurrent instability. As such, based on the results of this study, either anatomic SB or DB ACLR can be performed with a 10 mm-wide quadriceps tendon graft for individuals with ACL insertion sites that range from 14 to 18 mm.

## Supplementary Information

Below is the link to the electronic supplementary material.

Supplementary file1 (DOCX 19 KB)

## References

[1] Anderson AF, Irrgang JJ, Kocher MS (2006). International knee documentation committee subjective knee. Am J Sports Med.

[2] Ardern CL, Taylor NF, Feller JA, Webster KE (2014). Fifty-five per cent return to competitive sport following anterior cruciate ligament reconstruction surgery: an updated systematic review and meta-analysis including aspects of physical functioning and contextual factors. Br J Sports Med..

[3] Ardern CL, Webster KE, Taylor NF, Feller JA (2011). Return to sport following anterior cruciate ligament reconstruction surgery: a systematic review and meta-analysis of the state of play. Br J Sports Med..

[4] Biau DJ, Tournoux C, Katsahian S, Schranz P, Nizard R (2007). ACL reconstruction: a meta-analysis of functional scores. Clin Orthop Rel Res..

[5] Biau DJ, Tournoux C, Katsahian S, Schranz PJ, Nizard RS (2006). Bone-patellar tendon-bone autografts versus hamstring autografts for reconstruction of anterior cruciate ligament: meta-analysis. BMJ.

[6] Desai N, Bjornsson H, Musahl V (2014). Anatomic single- versus double-bundle ACL reconstruction: a meta-analysis. Knee Surg Sports Traumatol Arthrosc..

[7] Fithian DC, Paxton EW, Stone ML, Luetzow WFC, R.P., Daniel DM.  (2005). Prospective trial of a treatment algorithm for the management of the anterior cruciate ligament-injured knee. Am J Sports Med..

[8] Fu FH, Rabuck SJ, West RV, Tashman S, Irrgang JJ (2019). Patellar fractures after the harvest of a quadriceps tendon autograft with a bone block: a case series. Orthop J Sports Med..

[9] Getgood AMJ, Bryant DM, Litchfield R (2020). Lateral extra-articular tenodesis reduces failure of hamstring tendon autograft anterior cruciate ligament reconstruction: 2-year outcomes from the stability study randomized clinical trial. Am J Sports Med..

[10] Hussein M, van Eck CF, Cretnik A, Dinevski D, Fu FH (2012). Individualized anterior cruciate ligament surgery: a prospective study comparing anatomic single- and double-bundle reconstruction. Am J Sports Med..

[11] Hussein M, van Eck CF, Cretnik A, Dinevski D, Fu FH (2012). Prospective randomized clinical evaluation of conventional single-bundle, anatomic single-bundle, and anatomic double-bundle anterior cruciate ligament reconstruction: 281 cases with 3- to 5-year follow-up. Am J Sports Med..

[12] Irrgang JJ, Anderson AF, Boland AL (2006). Responsiveness of the international knee documentation committee subjective knee form. Am J Sports Med..

[13] Irrgang JJ, Enseki KR (2008) Rehabilitation following ACL reconstruction. In: Fu FH, Cohen S, eds. Current concepts in ACL reconstruction. New Jersey: Slack, Inc.; 377

[14] Kongtharvonskul J, Attia J, Thamakaison S (2013). Clinical outcomes of double- vs single-bundle anterior cruciate ligament reconstruction: a systematic review of randomized control trials. Scand J Med Sci Sports..

[15] Lamsam C, Kaewpornsawan K, Luangsa-Ard J (2012). Single-bundle versus double-bundle anterior cruciate ligament reconstruction: a meta-analysis. J Med Assoc Thai..

[16] Li RT, Lorenz S, Xu Y (2011). Predictors of radiographic knee osteoarthritis after anterior cruciate ligament reconstruction. Am J Sports Med..

[17] Li X, Xu CP, Song JQ, Jiang N, Yu B (2013). Single-bundle versus double-bundle anterior cruciate ligament reconstruction: an up-to-date meta-analysis. Int Orthop..

[18] Li YL, Ning GZ, Wu Q (2014). Single-bundle or double-bundle for anterior cruciate ligament reconstruction: a meta-analysis. Knee..

[19] Lohmander LS, Stenberg A, Englund M, Roos H (2004). High prevalence of knee osteoarthritis, pain, and functional limitations in female soccer players twelve years after anterior cruciate ligament injury. Arthritis Rheum.

[20] Mascarenhas R, Cvetanovich GL, Sayegh ET (2015). Does Double-bundle anterior cruciate ligament reconstruction improve postoperative knee stability compared with single-bundle techniques? a systematic review of overlapping meta-analyses. Arthroscopy.

[21] Meredick RB, Vance KJ, Appleby D, Lubowitz JH (2008). Outcome of single-bundle versus double-bundle reconstruction of the anterior cruciate ligament: A meta analysis. Am J Sports Med..

[22] Spindler KP, Huston LJ, Moon Knee Group (2018). Ten-year outcomes and risk factors after anterior cruciate ligament reconstruction: a moon longitudinal prospective cohort study. Am J Sports Med.

[23] Mouarbes D, Menetrey J, Marot V, Courtot L, Berard E, Cavaignac E (2019). Anterior cruciate ligament reconstruction: a systematic review and meta-analysis of outcomes for quadriceps tendon autograft versus bone-patellar tendon-bone and hamstring-tendon autografts. Am J Sports Med..

[24] Muller B, Yabroudi MA, Lynch A (2016). Defining thresholds for the patient acceptable symptom state for the IKDC subjective knee form and KOOS for patients who underwent ACL reconstruction. Am J Sports Med..

[25] Pombo MW, Shen W, Fu FH (2008). Anatomic double-bundle anterior cruciate ligament reconstruction: Where are we today?. Arthroscopy.

[26] Rothrauff BB, Jorge A, de Sa D, Kay J, Fu FH, Musahl V (2020). Anatomic ACL reconstruction reduces risk of post-traumatic osteoarthritis: a systematic review with minimum 10-year follow-up. Knee Surg Sports Traumatol Arthrosc..

[27] Sundemo D, Mårtensson J, Hamrin Senorski E (2019). No correlation between femoral tunnel orientation and clinical outcome at long-term follow-up after non-anatomic anterior cruciate ligament reconstruction. Knee Surg Sports Traumatol Arthrosc..

[28] Xu M, Gao S, Zeng C (2013). Outcomes of anterior cruciate ligament reconstruction using single-bundle versus double-bundle technique: meta-analysis of 19 randomized controlled trials. Arthroscopy.

