# Supplementary material for: Anatomic single vs. double-bundle ACL reconstruction: a randomized clinical trial–Part 1: clinical outcomes
Source: Knee Surg Sports Traumatol Arthrosc. 2021 May 10;29(8):2665–75. doi: 10.1007/s00167-021-06585-w (PMC8298248; doi:10.1007/s00167-021-06585-w)
Supplement: Supplementary file 1 — Supplementary file1 (DOCX 19 KB) [file 167_2021_6585_MOESM1_ESM.docx]

| **Supplemental Table 1 – Secondary Patient-Reported Outcome Measures** | | | | | |
| --- | --- | --- | --- | --- | --- |
|  | **Double Bundle**  **(n=29)** | **n** | **Single Bundle**  **(n=28)** | **n** | **P Value** |
| **KOS – ADLS** |  |  |  |  |  |
| Baseline | 68.0 ± 18.7 | 29 | 67.3 ± 19.9 | 28 | n.s*^a^* |
| 3 Months | 81.6 ± 10.6 | 28 | 85.2 ± 12.9 | 26 |  |
| 6 Months | 91.3 ± 6.0 | 28 | 91.0 ± 9.4 | 26 |  |
| 12 Months | 95.8 ± 4.0 | 27 | 90.5 ± 12.9 | 23 |  |
| 24 Months | 94.8 ± 7.1 | 27 | 95.0 ± 6.9 | 24 |  |
| **KOOS – Pain** |  |  |  |  |  |
| Baseline | 67.9 ± 14.4 | 29 | 69.4 ± 17.7 | 28 | n.s*^a^* |
| 3 Months | 82.7 ± 12.6 | 28 | 86.8 ± 11.5 | 26 |  |
| 6 Months | 91.1 ± 8.9 | 28 | 90.7 ± 10.5 | 26 |  |
| 12 Months | 94.6 ± 6.4 | 27 | 90.7 ± 12.2 | 23 |  |
| 24 Months | 95.8 ± 5.4 | 27 | 92.6 ± 12.8 | 24 |  |
| **KOOS – Other Symptoms** | |  |  |  |  |
| Baseline | 63.7 ± 16.1 | 29 | 59.3 ± 22.6 | 28 | n.s*^a^* |
| 3 Months | 73.0 ± 15.9 | 28 | 77.1 ± 13.8 | 26 |  |
| 6 Months | 81.3 ± 14.7 | 28 | 83.0 ± 14.4 | 26 |  |
| 12 Months | 87.0 ± 10.2 | 27 | 81.7 ± 15.5 | 23 |  |
| 24 Months | 89.3 ± 9.6 | 25 | 85.6 ± 9.2 | 23 |  |
| **KOOS – ADL** |  |  |  |  |  |
| Baseline | 78.7 ± 17.3 | 29 | 75.5 ± 17.9 | 28 | n.s*^a^* |
| 3 Months | 91.4 ± 8.6 | 28 | 93.7 ± 8.3 | 26 |  |
| 6 Months | 95.9 ± 6.3 | 28 | 96.2 ± 6.0 | 26 |  |
| 12 Months | 99.5 ± 1.1 | 27 | 95.7 ± 8.7 | 23 |  |
| 24 Months | 99.4 ± 1.8 | 27 | 97.4 ± 9.0 | 24 |  |
| **KOOS – Sports & Recreation** | |  |  |  |  |
| Baseline | 39.5 ± 24.3 | 29 | 41.4 ± 25.3 | 28 | n.s.*^a^* |
| 3 Months | 57.0 ± 26.0 | 28 | 68.7 ± 24.0 | 26 |  |
| 6 Months | 75.7 ± 20.0 | 28 | 75.2 ± 17.6 | 26 |  |
| 12 Months | 88.9 ± 11.9 | 27 | 86.1 ± 17.1 | 23 |  |
| 24 Months | 89.1 ± 13.4 | 27 | 90.4 ± 13.3 | 24 |  |
| **KOOS – Knee-Related QoL** | |  |  |  |  |
| Baseline | 34.7 ± 21.4 | 29 | 36.4 ± 19.9 | 28 | n.s*^a^* |
| 3 Months | 43.1 ± 15.9 | 28 | 50.0 ± 17.3 | 26 |  |
| 6 Months | 57.1 ± 19.4 | 28 | 58.4 ± 19.3 | 26 |  |
| 12 Months | 76.9 ± 16.8 | 27 | 70.1 ± 23.6 | 23 |  |
| 24 Months | 81.3 ± 20.9 | 27 | 81.8 ± 22.4 | 24 |  |
| **VR-12 Physical Components** | |  |  |  |  |
| Baseline | 39.9 ± 10.0 | 29 | 38.0 ± 9.1 | 28 | 0.02*^a^* |
| 3 Months | 41.2 ± 8.9 | 28 | 48.4 ± 8.3 | 26 |  |
| 6 Months | 46.9 ± 8.0 | 28 | 49.7 ± 6.0 | 26 |  |
| 12 Months | 51.9 ± 5.0 | 27 | 50.3 ± 7.9 | 23 |  |
| 24 Months | 54.1 ± 3.2 | 25 | 53.1 ± 4.6 | 23 |  |
| **VR-12 Mental Components** | |  |  |  |  |
| Baseline | 54.8 ± 7.1 | 29 | 52.3 ± 6.5 | 28 | n.s.*^a^* |
| 3 Months | 54.3 ± 6.8 | 28 | 52.9 ± 8.6 | 26 |  |
| 6 Months | 53.9 ± 6.6 | 28 | 52.6 ± 6.5 | 26 |  |
| 12 Months | 54.5 ± 5.3 | 27 | 54.8 ± 5.8 | 23 |  |
| 24 Months | 53.5 ±4.0 | 25 | 53.3 ± 6.1 | 23 |  |
| **AM-PAC Basic Mobility** |  |  |  |  |  |
| Baseline | 63.5 ± 6.9 | 28 | 65.1 ± 9.2 | 26 | n.s*^a^* |
| 3 Months | 66.3 ± 8.4 | 23 | 71.0 ± 9.9 | 17 |  |
| 6 Months | 74.2 ± 9.6 | 27 | 73.9 ± 8.7 | 23 |  |
| 12 Months | 80.4 ± 11.7 | 24 | 80.2 ± 10.8 | 21 |  |
| 24 Months | 84.8 ± 12.2 | 25 | 83.3 ± 11.1 | 24 |  |
| **ACL - RSI** |  |  |  |  |  |
| 12 Months | 58.5 ± 9.5 | 27 | 57.0 ± 9.7 | 23 | n.s.*^a^* |
| 24 Months | 59.2 ± 15.3 | 27 | 57.4 ± 8.0 | 24 |  |
| *^a^* P value for test of group by time interaction (fixed effects) controlling for intra-patient correlation (random effect) | | | | | |

| **Supplemental Table 2 – Range of Motion** |  |  |
| --- | --- | --- |
|  | **Double Bundle** |  |
| **3 Months Follow-up** | n=28 |  |
| Involved Knee Extension*^a^* | 2° ± 3° |  |
| Non-Involved Knee Extension*^a^* | 6° ± 3° |  |
| Side to Side Difference Knee Extension | 4° ± 3° |  |
| < 3° Difference in Knee Extension | 10 (35.7%) |  |
| Involved Knee Flexion | 139° ± 8° |  |
| Non-Involved Knee Flexion | 145° ± 5° |  |
| Side to Side Difference Knee Flexion | 6° ± 8° |  |
| < 5° Difference in Knee Flexion | 18 (64.3%) |  |
| **6 Months Follow-up** | n=28 |  |
| Involved Knee Extension*^a^* | 3° ± 4° |  |
| Non-Involved Knee Extension*^a^* | 7° ± 3° |  |
| Side to Side Difference Knee Extension | 4° ± 4° |  |
| < 3° Difference in Knee Extension | 16 (57.1%) |  |
| Involved Knee Flexion | 142° ± 7° |  |
| Non-Involved Knee Flexion | 144° ± 5° |  |
| Side to Side Difference Knee Flexion | 2° ± 6° |  |
| < 5° Difference in Knee Flexion | 25 (89.3%) |  |
| **12 Months Follow-up** | n=27 |  |
| Involved Knee Extension*^a^* | 4° ± 3° |  |
| Non-Involved Knee Extension*^a^* | 6° ± 3° |  |
| Side to Side Difference Knee Extension | 2° ± 2° |  |
| < 3° Difference in Knee Extension | 20 (70.4%) |  |
| Involved Knee Flexion | 143° ± 5° |  |
| Non-Involved Knee Flexion | 143° ± 6° |  |
| Side to Side Difference Knee Flexion | 0° ± 4° |  |
| < 5° Difference in Knee Flexion | 24 (88.9%) |  |
| **24 Months Follow-up** | n=27 |  |
| Involved Knee Extension*^a^* | 4° ± 3° |  |
| Non-Involved Knee Extension*^a^* | 6° ± 3° |  |
| Side to Side Difference Knee Extension | 2° ± 2° |  |
| < 3° Difference in Knee Extension | 19 (70.4%) |  |
| Involved Knee Flexion | 143° ± 5° |  |
| Non-Involved Knee Flexion | 144° ± 5° |  |
| Side to Side Difference Knee Flexion | 1° ± 3° |  |
| < 5° Difference in Knee Flexion | 26 (96.3%) |  |
| *^a^* Positive knee extension values indicate degrees of hyperextension | | |
